# Development of a classifier for [18F]fluorodeoxyglucose extravasation severity using semi-quantitative readings from topically applied detectors

**DOI:** 10.1186/s40658-022-00488-6

**Published:** 2022-09-14

**Authors:** Steve Perrin, Jackson W. Kiser, Josh Knowland, Spencer L. Bowen

**Affiliations:** 1 Lucerno Dynamics LLC, Cary, NC USA; 2 Department of Radiology, Carilion Clinic, Roanoke, VA USA; 3 Department of Radiology, UT Southwestern Medical Center, Dallas, TX USA

**Keywords:** Extravasation, PET/CT, Classification, Quantification, Quality control

## Abstract

**Background:**

Radiotracer extravasations, caused largely by faulty tracer injections, can occur in up to 23% of ^18^F-fluorodeoxyglucose (FDG) PET/CT scans and negatively impact radiological review and tracer quantification. Conventional radiological assessment of extravasation severity on PET has limited performance (e.g., extravasations frequently resolve before scanning) and practical drawbacks. In this study, we develop a new topical detector-based FDG extravasation severity classifier, calibrated from semi-quantitative PET measurements, and assess its performance on human subjects.

**Methods:**

A retrospective study examined patients whose FDG injections had been monitored as part of their standard workup for PET/CT imaging. Topical uncollimated gamma ray detectors were applied proximal to the injection site and on the same location on the opposing arm, and readings were acquired continuously during radiotracer uptake. Patients were imaged with their arms in the PET field of view and total extravasation activity quantified from static PET images through a volume of interest approach. The image-derived activities were considered ground truth and used to calibrate and assess quantification of topical detector readings extrapolated to the start of PET imaging. The classifier utilizes the calibrated detector readings to produce four extravasation severity classes: none, minor, moderate, and severe. In a blinded study, a radiologist qualitatively labeled PET images for extravasation severity using the same classifications. The radiologist’s interpretations and topical detector classifications were compared to the ground truth PET results.

**Results:**

Linear regression of log-transformed image-derived versus topical detector tracer extravasation activity estimates showed a strong correlation (*R*^2^ = 0.75). A total of 24 subject scans were cross-validated with the quantitatively based classifier through a leave-one-out methodology. For binary classification (none vs. extravasated), the topical detector classifier had the highest overall diagnostic performance for identifying extravasations. Specificity, sensitivity, accuracy, and positive predictive value were 100.0%, 80.0%, 95.8%, and 100.0%, respectively, for the topical detector classifier and 31.6%, 100.0%, 45.8%, and 27.8%, respectively, for the radiological analysis. The topical detector classifier, with an optimal detection threshold, produced a significantly higher Matthews correlation coefficient (MCC) than the radiological analysis (0.87 vs. 0.30).

**Conclusions:**

The topical detector binary classifier, calibrated using quantitative static PET measurements, significantly improves extravasation detection compared to qualitative image analysis.

**Supplementary Information:**

The online version contains supplementary material available at 10.1186/s40658-022-00488-6.

## Background

Radiotracer extravasations can negatively impact both radiological review and tracer quantification for FDG PET/CT. Characterized by retention of tracer proximal to the injection site, extravasations have been reported to occur at a frequency of 1–23% [1, 2] and as high as 50% of the injected dose [3]. The most commonly utilized semi-quantitative radiotracer uptake measure, the standardized uptake value (SUV), can be significantly biased due to extravasations. A tracer extravasation can effectively delay tissue tracer equilibrium, after a standard bolus injection, and reduce venous tracer delivery. Both of these factors may increase SUV bias, as the SUV assumes imaging at tracer transient equilibrium and injection of the total activity into the venous system. Osman et al. [4] measured a greater than 9% deficit in liver and mediastinum max SUVs between repeat scans of subjects with tracer extravasations relative to extravasation-free scans. Kiser et al. [5] noted an increase in max SUV from 24 to 43 for a lung lesion in PET scans of the same subject with a severe extravasation and normal injection, respectively. The quantitative imaging biomarker alliance (QIBA) emphasizes the impact of tracer extravasations on PET quantification in their guidelines for maximizing PET performance in clinical studies of therapy response monitoring [6]. Specifically, the guidelines recommend estimating extravasation activity from PET images, and if exceeding 5% of the injected activity, potentially disqualifying the data point from the study review. Qualitatively, a relatively high activity extravasation can produce image reconstruction artifacts that obscure both normo- and pathophysiological uptake [5].

Radiological analysis of extravasation severity during static PET imaging suffers from both performance and practical drawbacks. On the performance side, extravasations frequently resolve before imaging. For example, Lattanze et al. [7] found that 10 of 17 cases with FDG extravasations detected on dynamic PET were not visible after the standard ~ 60-min uptake period. Furthermore, extravasation kinetics cannot be accurately evaluated from a post-uptake static PET image. Thus, a comparatively high uptake extravasation with rapid resolution and a relatively low uptake extravasation with slower resolution may appear similar post-uptake, but produce substantial differences in SUV bias compared to the extravasation-free case. Practical drawbacks of estimating extravasation severity through radiological analysis are as follows: (1) the radiologist and/or technologist will need to devote additional time for each image series to assess extravasation severity, on top of the standard-of-care reading, and (2) PET image quality and quantification of the torso is typically better when the subject’s arms are positioned above the head [8], such that the injection and extravasation location is frequently not in the field of view (FoV). For example, a recent study [9] found that 37% of extravasations were not in the PET FoV for general FDG PET/CT imaging. In addition, focused examinations (e.g., brain studies) typically do not image the injection site.

A topical detector system for measuring the presence of radiopharmaceutical in a body region is listed as a Class I exempt device by the FDA and has been evaluated in several clinical studies [1]. The Lara® System (Lucerno Dynamics, LLC) utilizes two 511 keV gamma ray detectors, with one detector placed proximal to the injection site and the other positioned in a matched location on the contralateral arm. Time-activity curves (TACs) of photon count rates are then recorded during tracer injection and throughout most of the uptake period. The difference between the TACs recorded from the two detectors helps clinicians assess the presence of localized radiotracer prior to imaging and evaluate its biological clearance. A multicenter quality improvement study demonstrated that the use of the topical detector system enabled a significant reduction in the frequency of extravasations (from 8.9 to 4.6%) [1]. Topical detector classification of extravasation severity has been developed through radiological analysis of dynamic PET scans [7] and agreed with the radiological interpretations in 86% of subjects in one study. However, the use of quantitative measures from the topical detector system, to improve extravasation severity classification, has not been evaluated.

Our objective is to develop a new topical detector-based FDG extravasation severity classifier, calibrated from static semi-quantitative PET measurements, and assess its performance on human subjects. Motivated by the benefits of semi-quantitative PET imaging for oncological therapy response assessment and prediction [10, 11], we aimed to explore how semi-quantification of extravasation activity with the topical detector system can impact classification. The classifier leverages extravasation severity activity thresholds proposed by QIBA in their technically confirmed profile for maximizing PET performance in clinical studies of therapy response monitoring [6]. The diagnostic performance of the resulting topical detector classifier is compared against ground truth PET estimates and qualitative classification by a radiologist.

## Methods

### Overview

Development and evaluation of the extravasation severity classifier, that inputs topical detector readings, were performed with a retrospective human subject study. Subjects included those monitored with the topical detector system, during tracer uptake, and then scanned with FDG PET/CT (“Patient data” section). The topical detector system is detailed in the “Extravasation severity topical detector” section. Ground truth extravasation activity was measured from static PET scans using a volume of interest (VOI) approach  (“PET-based extravasation activity estimation” section). Due to the removal of topical detectors a median of 15 min before PET/CT, we utilized topical detector count rates extrapolated to the start of PET imaging (“Topical detector count rate extrapolation” section), enabling a direct comparison of static PET and topical detector readings. The extravasation severity classifier is described in “Semi-quantitative topical detector extravasation severity classifier” section and uses a regression of ground truth PET measurements against topical detector readings to produce semi-quantitative estimates of extravasation activity from the latter. Extravasation severity classification performance of the topical detector classifier was compared against a qualitative review by a radiologist and the ground truth PET values (“Radiological comparison study” section).

### Patient data

The retrospective study examined patients whose FDG injections had been monitored with the topical detector system, from the time of injection until just prior to imaging, as part of their standard clinical workup for PET/CT. This study was approved by the Carilion Clinic Institutional Review Board (IRB), and the need for written informed consent was waived by the IRB. This retrospective study included a total of 24 subjects, with injection activity and uptake time of 320 ± 86 MBq and 68.0 ± 13.7 min (mean ± *σ*), respectively. Due to the use of de-identified datasets, patient gender and age were not available in this retrospective review. However, no exclusion criteria based on gender were utilized, and we only excluded vulnerable populations less than 18 or greater than 89 years old, for subject data collection. The study population contains subjects selected to represent a range of extravasation severity levels, identified through a qualitative assessment of topical detector TACs. Additionally, selected subjects had been imaged with the injection location (i.e., antecubital fossa, hand, etc.) positioned within the PET FoV.

The technologist utilized standard practice for selecting the injection location (e.g., preferentially inject in the arm contralateral to the primary cancer). When possible, subjects were injected with an infusion system (MEDRAD Intego, Bayer), with the infusion lasting ~ 1–2 min and included periods of saline flushing. In cases where the infusion system was not available, the dose was administered manually in ~ 1–10 s., followed by a saline flush. During the injection process and monitoring with the topical detector system, patients were positioned in an upright uptake chair with arms placed at their side on armrests. Subjects were asked to avoid excessive movement, but were allowed to move their arms as necessary during uptake.

Subjects were scanned on a Siemens Biograph mCT, with FlowMotion, or a Biograph TruePoint PET/CT (Siemens Medical Solutions USA, Inc.) and images reconstructed based on standard of care. Importantly, the Biograph mCT has a time-of-flight-capable PET camera, while the Biograph TruePoint does not. For both scanners, the imaging protocol began with a topogram, CT acquisition (120 kV and variable mA), and PET scan covering the subject from the skull base to the upper thighs. For the Biograph mCT, the PET acquisition utilized a continuous bed motion (table speed ranging from 0.7 to 1.6 mm/s., step-and-shoot equivalent of 180 and 80 s./bed, respectively), and images were reconstructed with time-of-flight ordered-subsets expectation maximization (OSEM) using 2 iterations and 21 subsets. For patients imaged on the Biograph TruePoint PET/CT, multiple bed positions with a step-and-shoot protocol were used to cover the subject with a scan duration of 3 min each (5 min for subjects above 113 kg), and images were reconstructed with OSEM using 4 iterations and 8 subsets. For both systems, reconstructed images were fully corrected for all factors impacting quantification (e.g., attenuation, scatter, etc.), sampled at 4.1 × 4.1 mm in axial slices, and smoothed in post-reconstruction with a 5-mm FWHM Gaussian filter. Slice thicknesses were 5.0 and 3.0 mm, for the Biograph mCT and Biograph TruePoint images, respectively.

### Extravasation severity topical detector

**Fig. 1:**
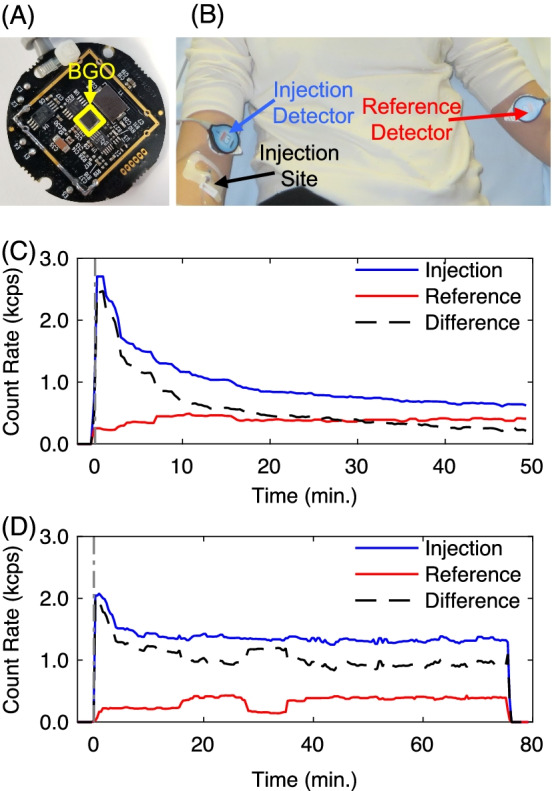
Overview of the topical detector system used for classifying extravasation severity. **A** Single detector electronics that include a BGO scintillator coupled to a silicon photomultiplier and (**B**) placement of the two system detectors on the subject’s injection and contralateral arms; injection and reference detectors, respectively. **C**, **D** Representative TACs demonstrating signals from the injection and reference detectors, and their difference for subjects (**C**) with negligible and (**D**) moderate extravasation

An overview of the topical detector system, as well as sample datasets, is shown in Fig. 1. The system consists of two 511 keV gamma ray detectors. Complete specifications and performance measurements (e.g., count rate and energy linearity) of the system have been detailed by Knowland et al. [12]. Each detector is constructed of a monolithic 3 × 3 × 3 mm bismuth germanate (BGO) scintillator coupled to a silicon photomultiplier. No attenuative collimation is employed to restrict gamma ray incident angles. For readout electronics, a lower level energy discriminator of ~ 504 keV is applied and gamma ray flux (cps) is output for each detector at 1 Hz. Importantly, each detector measures and outputs photon count rates independently (i.e., no coincidence detection between detectors is utilized). Error in count rate linearity for a single topical detector for ^18^F was ≤ 1.5%, up to a true count rate of 80 kcps [12]. Both detectors are adhesively attached to the patient’s arms prior to radiotracer injection, with one detector (injection detector) placed proximal to the injection site and the other (reference detector) positioned in a matched location on the contralateral arm. TACs are then recorded continuously beginning immediately prior to tracer injection and during nearly the full uptake period. The main operating assumption is that the background signal from the body and arms is comparable in both detectors, such that the difference in TACs is primarily due to excess activity from an extravasation in the injection arm (Fig. 1C, D). We note that this operating assumption has been validated with a human subjects study comparing dynamic PET scans (ground truth) against topical detector readings [7]. In the absence of extravasations, TACs between injection and reference detectors matched closely. We provide additional support for this operating assumption here through a Monte Carlo simulation using a digital anthropomorphic phantom (see Additional file 1). TACs are corrected for tracer decay. No correction for solid angle, scattered photons, or photon attenuation is applied.

### Data processing

#### PET-based extravasation activity estimation

**Fig. 2:**
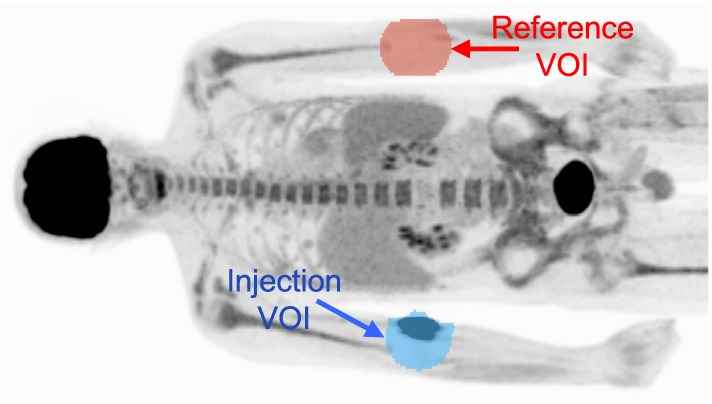
Representative VOI placement for the PET-based extravasation activity estimation method. VOIs were placed on the injection and contralateral arms, “Injection VOI” and “Reference VOI,” respectively

Ground truth estimates of total extravasation activity were measured from PET images using a VOI-based approach adapted from that of Silva-Rodríquez et al. [13]. Noting the recorded injection location (e.g., right hand), both the tissue surrounding the injection (injection arm) and matched anatomical area of the contralateral arm (reference arm) were manually “painted” with initial VOIs on axial PET image slices using 3D Slicer [14]. An example of these VOIs is shown in Fig. 2. Initial VOIs were deliberately drawn to exceed the qualitative boundaries of both extravasation and normal physiological uptake. Total activity in the injection area ($I_{{{\text{TOT}}}}$), above what would be expected with normal physiological uptake alone, was computed as follows:

1 $$I_{{{\text{TOT}}}} = V_{{\text{V}}} \left[ {\mathop \sum \limits_{i} m_{{{\text{I}},i}} x_{i} - \frac{{V_{{\text{I}}} }}{{V_{{\text{R}}} }}\mathop \sum \limits_{i} m_{{{\text{R}},i}} x_{i} } \right]$$

where $V_{{\text{V}}}$ is the reconstructed image voxel volume, $m_{{\text{I}}}$ and $m_{{\text{R}}}$ are the binary masks for the injection and reference arms, respectively, $x_{i}$ is the PET image in units of Bq/ml, and $V_{{\text{I}}}$ and $V_{{\text{R}}}$ are total volumes for the injection and reference binary VOIs, respectively. Both $m_{{\text{I}}}$ and $m_{{\text{R}}}$ were produced from the original VOIs by imposing an SUV threshold of 0.2 to mitigate the impact of noisy background voxels.

#### Topical detector count rate extrapolation

The difference of topical detector count rates was extrapolated from the time of detector removal to the start of PET imaging to enable a direct comparison against static ground truth PET extravasation activity estimates. Topical detectors were removed a median of 15 min before the start of PET imaging to allow time for the patient to void and for positioning in the PET scanner. Thus, besides the purpose of extrapolation, we do not use the dynamic information in topical detector TACs for extravasation severity classification in this study. The extrapolation method proceeded as follows: (1) take the difference of injection and reference arm TACs, (2) increase the extrapolation time start point past early segments, with large baseline transitions, utilizing a gradient-based approach, and (3) employ robust time-shifted decaying exponential function regression with the bisquare weights method and fit weights ($$W_{i} )$$ calculated as:

2 $$W_{i} = t_{i} - t_{s} + 1$$

where $t_{i}$ is the sample time, for sample *i*, and $t_{s}$ the fit start time. Nonlinear least squares regression was performed on up to 20 min of data and as little as 8 min, depending on the length of the TAC and the adjusted fit start time. We refer to the difference in topical detector count rates, extrapolated to the start of PET imaging, as $\hat{D}$. The performance of the extrapolation method was assessed using a total of 23 relatively long (time $\ge$ 40 min) topical detector TACs taken from a subset of the patient datasets described in the “Patient data” section. TACs were synthetically clipped by 15 min and extrapolated to the artificial end time. Ground truth count rates were calculated from the difference of the median injection and reference TACs taken over 3 min.

### Semi-quantitative topical detector extravasation severity classifier

A simple decision-tree-based multiclass extravasation severity classifier, inputting topical detector measurements, was derived from the PET ground truth extravasation activity values. For developing the classifier and assessing the general quantification of topical detector measurements, we performed the following steps: (1) topical detector difference readings were extrapolated to the start of PET imaging ($\hat{D}$ in the “Topical detector count rate extrapolation” section) and adjusted for radionuclide decay and positron fraction, and (2) PET image extravasation activities ($I_{{{\text{TOT}}}}$ in Bq) were correlated against the extrapolated topical values ($\hat{D}$ in cps) with two different linear regression strategies, enabling estimation of total extravasation activity ($\hat{I}_{{{\text{TOT}}}}$) from topical detector count rates alone. Equations for the least squares regression models were as follows:

3 $$\hat{I}_{{{\text{TOT}},{\text{Lin}}}} = b_{1} \hat{D}$$

4 $$\ln \left( {\hat{I}_{{{\text{TOT}},{\text{Log}}}} } \right) = b_{0} + b_{1} \ln \left( {\hat{D} } \right)$$

where $\hat{I}_{{{\text{TOT}},{\text{Lin}}}}$ and $\hat{I}_{{{\text{TOT}},{\text{Log}}}}$ are estimates of total extravasation activity from the two models, $b_{0}$ and $b_{1}$ are offset and slope parameters, and $\ln ()$ is the natural logarithm. Thus, (4) is a linear regression on log-transformed PET image extravasation activities and extrapolated topical count rates. Ordinary least squares regression was used to estimate parameters for both models. For convenience, we refer to (3) and (4) as linear and log fits, respectively.

Normalizing the estimated total extravasation activity ($\hat{I}_{{{\text{TOT}}}}$) by the decay compensated injected dose ($A_{{{\text{INJ}}}}$) produces the fraction of activity in the tracer extravasation relative to the total injected activity ($\hat{I}_{{{\text{TOT}}}} /A_{{{\text{INJ}}}}$). A total of four classes was then used to stratify normalized extravasation activity as follows: (1) none ($\hat{I}_{{{\text{TOT}}}} /A_{{{\text{INJ}}}}$ < 1%), (2) minor (1% ≤ $\hat{I}_{{{\text{TOT}}}} /A_{{{\text{INJ}}}}$ < 5%), (3) moderate (5% ≤ $\hat{I}_{{{\text{TOT}}}} /A_{{{\text{INJ}}}}$ < 20%), and (4) severe (20% ≤ $\hat{I}_{{{\text{TOT}}}} /A_{{{\text{INJ}}}}$). QIBA guidelines [6] specify $\hat{I}_{{{\text{TOT}}}} /A_{{{\text{INJ}}}}$ < 5% as minor and match the $\hat{I}_{{{\text{TOT}}}} /A_{{{\text{INJ}}}}$ moderate and severe classes given above. We rationalize the lowest threshold as follows: (1) static PET images provide no information on tracer extravasation resolution kinetics and (2) our prior experience with the qualitative impact of extravasations on PET images.

To evaluate the overall quantification of topical detector differences, the linear regression analysis of $I_{{{\text{TOT}}}}$ versus $\hat{D}$ was performed on all human subjects data. For classification performance, a leave-one-out cross-validation was utilized to produce $\hat{I}_{{{\text{TOT}}}}$ values. We note that only the log fit, described by (4), was used to estimate total extravasation activity for assessing classification performance (see justification in the “Topical detector extravasation activity semi-quantification” section).

### Radiological comparison study

The performance of the semi-quantitative topical detector extravasation severity classifier was compared against the qualitative assessment of a nuclear medicine radiologist (J.W.K) with more than 25 years of experience. Extravasation severity is currently evaluated qualitatively in the clinic, with no standardized methodology available. Thus, this approach represents the clinically utilized and preferred scheme of the associated nuclear medicine radiologist for classifying extravasations. PET images were ordered randomly with respect to patient ID, de-identified of patient information, and reviewed individually on a syngo.via workstation (Siemens Medical Solutions USA, Inc.). Images evaluated included maximum intensity projections (MIPs) and axial, coronal, and sagittal slices. The injection location (e.g., right hand) for each subject was accessible by the radiologist. Extravasation severity was scored on a scale of 0–3 as follows: 0 (none) indicated no visible evidence of radiotracer at or around the injection site, a score of 1 (minor) demonstrated small abnormal accumulation visible at or near the injection site that did not cause visible degradation of regional anatomy or image quality, 2 (moderate) showed more extensive visual evidence of extravasation at or near the injection site causing image distortion, blurring or loss of resolution of the regional anatomy or structures in proximity to the injection site, and 3 (severe) indicated a very large radiotracer extravasation that completely obscures regional anatomy such that no structures are recognizable. Additionally, in the arms down position, severe extravasations were those that degraded image quality of adjacent internal structures such as the liver, spleen, or kidneys and caused image windowing limitations due to scaling problems.

A binary classification analysis was performed by labeling all extravasations measured on PET with an uptake of $I_{{{\text{TOT}}}} /A_{{{\text{INJ}}}}$ ≥ 5% as positives. For the topical detector classifier and radiological analysis, labels of moderate and severe were deemed positives. Performance was evaluated for this binary classification with the Matthews correlation coefficient (MCC). We also determined the optimal extravasation activity threshold ($\hat{I}_{{{\text{TOT}}}} /A_{{{\text{INJ}}}}$), for topical detector measurements alone, to maximize binary diagnostic performance.

### Statistical analysis

A percentile bootstrap method [15] (10,000 random samples) was utilized to estimate 95% confidence intervals of coefficients of determination from linear regression strategies. A Student’s *t*-test was used to test the significance between topical detector and radiological analysis binary classification results. Standard deviation was the result of a jackknifed standard error calculation. A *P* value of < 0.05 was deemed significant.

## Results

### Topical detector count rate extrapolation validation

**Fig. 3:**
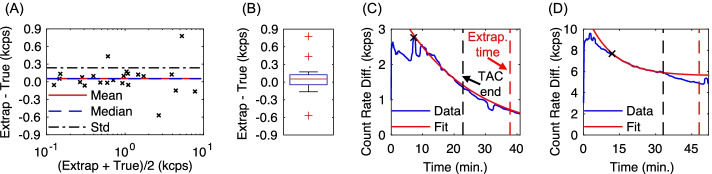
Validation of the topical detector TAC extrapolation method, assuming 15 min gap between the end of the TAC and the extrapolation time point. **A** Bland–Altman plot of extrapolated ($\hat{D}$) and ground truth measurements. **B** Box plot of differences between extrapolated and ground truth measurements, with central line indicating the median, box bottom and top edges representing the 25th and 75th percentiles, respectively, whiskers indicating the extremes, and red crosses indicating outliers. **C**, **D** Representative fits for cases with (**C**) minimal count rate bias and (**D**) outlier count rate bias due to a baseline shift after the assumed TAC end time, with automated fit start time indicated by “x” marks in both cases

A performance evaluation of the topical detector count rate extrapolation algorithm is shown in Fig. 3. In general, the extrapolation method showed both high accuracy and precision. The mean and standard deviation of paired differences (Fig. 3A) were 0.05 and 0.24 kcps, respectively, with 95% confidence intervals of [−0.41, 0.52] kcps. Three outliers were identified in the box plot, with the cause in all cases due to large TAC baseline shifts after the synthetic TAC cutoff time. Figure 3D shows an instance of such an outlier, resulting in a bias of 0.78 kcps (16% relative to the true count rate difference). The remaining two outliers had biases of 0.43 and − 0.57 kcps.

### Topical detector extravasation activity semi-quantification

**Fig. 4:**
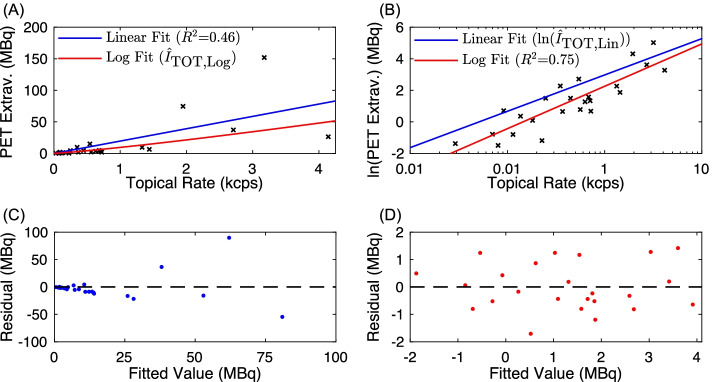
Correlation analysis of ground truth PET image extravasations ($I_{{{\text{TOT}}}}$) versus extrapolated difference in topical detector count rates ($\hat{D}$). Both linear and log fits, as detailed in (3) and (4), are shown. Plots depict (**A**) original and (**B**) log-transformed axes, with measured data denoted by black "x" marks. Residual fits for the (**C**) linear and (**D**) log fits, with zero residual marked by the dashed black line

Quantitative performance of the topical detectors for measuring total extravasation activity is shown in Fig. 4, including results for both the linear and log fits. Using all available subject data for the linear fit described in (3) produced a coefficient of determination (*R*^2^) of 0.46 (95% confidence interval of 0.31 to 0.85) and line with formula $\hat{I}_{{{\text{TOT}},{\text{Lin}}}} = 19.6 \hat{D}$. This *R*^2^ value is interpreted as a moderate strength linear correlation between ground truth extravasation activity ($I_{{{\text{TOT}}}}$) and topical detector difference count rates ($\hat{D}$) [16]. However, the residual plot of the linear fit ($I_{{{\text{TOT}}}} - \hat{I}_{{{\text{TOT}},{\text{Lin}}}}$ versus $\hat{I}_{{{\text{TOT}},{\text{Lin}}}} )$ (Fig. 4C) demonstrated a qualitative increase in noise as a function of estimated extravasation activity, which is a characteristic of heteroscedasticity. Ordinary least squares regression assumes independently and identically distributed residuals. Thus, when violated, heteroscedasticity can result in ill-specified variances of extravasation activity estimates and unstable coefficient (i.e., intercept and slope) estimations, ultimately negatively impacting classification performance. Applying log transformation to one or both axes is a common approach to correct for heteroscedasticity. Parameters for the log fit, described in (4), were 8.0 and 1.2 for $b_{0}$ and $b_{1}$, respectively. Log fit results (Fig. 4B) demonstrated a higher *R*^2^ = 0.75 (95% confidence interval of 0.58 to 0.86) than the linear fit, interpreted as a strong correlation between log-transformed $I_{{{\text{TOT}}}}$ versus $\hat{D}$ [16]. In addition, residual plots (Fig. 4D) demonstrated qualitatively similar variances throughout the range of $\ln \left( {\hat{I}_{{\text{TOT,Log}}} } \right)$ values, suggesting resolution of heteroscedasticity. Due to its higher performance, we used the log fit regression model exclusively for the leave-one-out analysis, a component of the classification study.

**Fig. 5:**
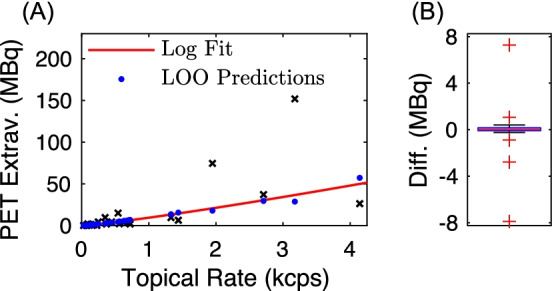
Performance of extravasation activity predictions from the leave-one-out (LOO) analysis with the log fit regression model. **A** LOO predictions and log fit results using all the measured data, with measured data denoted by black "x" marks. **B** Box plot of differences in extravasation activity predictions from the LOO analysis and those from the log fit results, using all the measured data

**Fig. 6:**
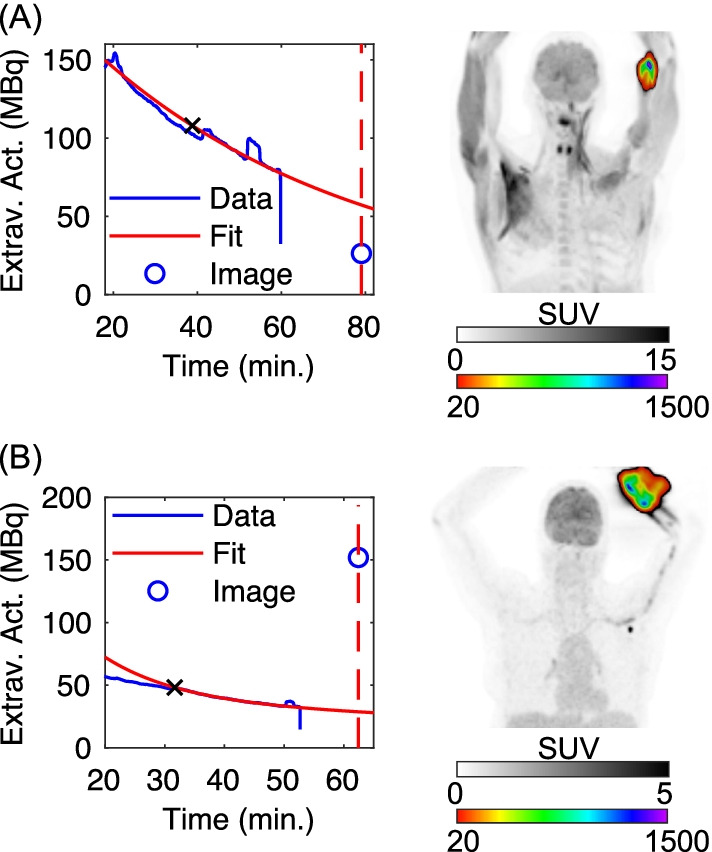
Topical detector TACs and PET images for two outliers in the leave-one-out analysis. Subjects with (**A**) left antecubital fossa ($I_{{{\text{TOT}}}}$ = 26.3 MBq and $\hat{D}$ = 4.1 kcps on Fig. 4A) and (**B**) left forearm ($I_{{{\text{TOT}}}}$ = 152 MBq and $\hat{D}$ = 3.2 kcps on Fig. 4A) injections, respectively. Topical detector TACs (“Data”) are scaled to total extravasation activity to produce $\hat{I}_{{{\text{TOT}}}}$, using the matched leave-one-out calibration factor. Extrapolation fits (“Fit”) (fit starts denoted by x’s) and ground truth PET extravasation activities, $I_{{{\text{TOT}}}}$ (“Image”), are also displayed. Coronal maximum intensity projections are shown in two color scales to better visualize distribution of tracer extravasations

Evaluation of extravasation activity predictions from the leave-one-out (LOO) analysis with the log fit regression model is shown in Fig. 5. A box plot (Fig. 5B) of the difference of predictions from the LOO regression and that from the log fit using all patient data demonstrated a median difference of 0 MBq and 25th and 75th percentiles of −0.1 and 0.2 MBq, respectively. Outliers were observed when leaving out comparatively high image-derived extravasation activities or topical detector estimates. For instance, leaving out the highest recorded PET-derived extravasation ($I_{{{\text{TOT}}}}$ = 152 MBq, $\hat{D}$ = 3.2 kcps) produced an outlier with a difference of −7.9 MBq. Additionally, leaving out the data point with $I_{{{\text{TOT}}}}$ = 26.3 MBq generated an overestimation in the LOO prediction of 7.3 MBq. The remaining three outlier LOO predictions were within ± 2.8 MBq of the log fit with all data. Example topical detector TACs and images for these outliers are shown in Fig. 6.

### Radiological comparison study

#### Multiclass classification performance

Multiclass classification results for the topical detector classifier and radiological analysis are shown in Tables 1 and 2, respectively. Due to the limited number of samples in each class, we do not compute multiclass performance metrics. However, based on confusion matrix values, the topical detector classifier and radiologist both had limited performance, with radiological review performance comparatively lower. Specifically, the topical detector classifier tended to have limited ability to predict “minor” ground truth extravasations, while the radiologist tended to overestimate extravasation severity by one or more classes for both the ground truth “none” and “minor” classes. Additionally, the topical detector classifier downgraded the class of the two true “severe” extravasations to “moderate.”

**Table 1:** Multiclass confusion matrix of extravasation severity for the topical detector classifier

Class predicted	Ground truth class (from PET measurements)			
	None	Minor	Moderate	Severe
None	9	2	0	0
Minor	4	2	1	0
Moderate	0	2	1	2
Severe	0	0	1	0

**Table 2:** Multiclass confusion matrix of extravasation severity for the radiological analysis

Class predicted	Ground truth class (from PET measurements)			
	None	Minor	Moderate	Severe
None	0	0	0	0
Minor	6	0	0	0
Moderate	3	4	2	0
Severe	4	2	1	2

#### Binary classification performance

Binary classification results for the topical detector classifier and radiological analysis are shown in Table 3. For the topical detector classifier alone, we utilized the standard threshold of $\hat{I}_{{{\text{TOT}}}} /A_{{{\text{INJ}}}}$ = 5% (QIBA threshold) and found an optimal threshold range of 6.9% < $\hat{I}_{{{\text{TOT}}}} /A_{{{\text{INJ}}}} < 7.2\%$ for detecting extravasations. Note that a 5% threshold is equivalent to grouping severe and moderate classes for positive cases, as detailed in the “Semi-quantitative topical detector extravasation severity classifier” section. Overall, the topical detector classifier had the highest diagnostic performance for identifying extravasations. The specificity, accuracy, and PPV of the topical detector classifier exceeded radiological analysis by ≥ 39% for the QIBA threshold and by ≥ 50% for the optimal threshold. The sensitivity of the topical detector classifier was 20% lower than the radiologist in both cases. Critically, the topical detector classifier produced a Matthews correlation coefficient (MCC) of 0.65 and 0.87 for the QIBA and optimal thresholds, respectively, which are both significantly higher (*P* < 0.05) than the radiological analysis (0.30). The MCC is a more robust measure of performance over classical diagnostic tests (e.g., sensitivity) given the comparatively low number of positive, relative to negative, cases in this study [17].

**Table 3:** Binary classification performance of the topical detectors and radiological analysis

Diagnostic metric	Topical detectors (QIBA threshold)	Topical detectors (optimal threshold)	Radiologist
TP	4	4	5
TN	17	19	6
FP	2	0	13
FN	1	1	0
Sensitivity (%)	80.0	80.0	100.0
Specificity (%)	89.5	100.0	31.6
Accuracy (%)	87.5	95.8	45.8
PPV (%)	66.7	100.0	27.8
NPV (%)	94.4	95.0	100.0
MCC	0.65*	0.87*	0.30

*Significantly different from the radiologist metric based on the jackknife estimate of standard error and Student’s *t-*test

The primary cause of the performance difference between topical and radiological classifiers is attributed to overdiagnosis by the radiologist, indicated by the at least eleven extra false positives relative to the topical detector classifications. There was only one instance where the topical detector classifier underperformed the radiologist; a positive ground truth 15.1 MBq ($I_{{{\text{TOT}}}} /A_{{{\text{INJ}}}}$ = 6.8%) extravasation, with topical detector activity estimated at $\hat{I}_{{{\text{TOT}}}} /A_{{{\text{INJ}}}}$ = 2.0%, resulting in a false negative. Both the radiologist and topical detector classifiers (QIBA threshold alone) produced false positives for the same two subjects: 2.8% and 3.7% $I_{{{\text{TOT}}}} /A_{{{\text{INJ}}}}$ image-derived extravasations. The topical detector classifier using the optimal threshold range (6.9% < $\hat{I}_{{{\text{TOT}}}} /A_{{{\text{INJ}}}} < 7.2\%$) eliminated both false positives, with topical detector extravasation activities measured at 5.3% and 6.9%, while not changing the lowest activity true positive ($\hat{I}_{{{\text{TOT}}}} /A_{{{\text{INJ}}}}$ = 7.2%). Thus, MCC increased by 0.22 for the optimal range versus QIBA threshold results.

**Fig. 7:**
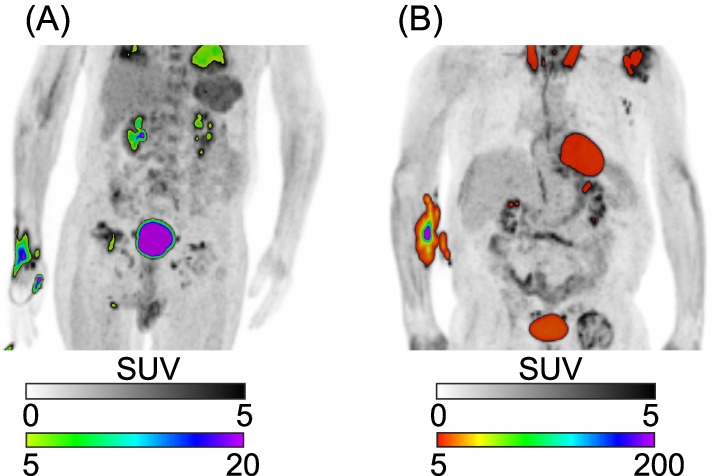
PET images for two noteworthy cases in the binary classification analysis. Subjects with (**A**) right-hand injection and (**B**) right forearm injections, respectively. Coronal maximum intensity projections are shown in two color scales to better visualize distribution of tracer extravasations

Example images of noteworthy cases from the binary classification analysis are shown in Fig. 7. A subject with a right-hand injection (Fig. 7A) was labeled a false positive on the radiological analysis, but a true negative with the topical detector classifier. A negative contrast artifact distal to the tracer extravasation was the primary justification for the positive radiological classification. A subject with a right forearm injection was a false positive on both the radiological and topical ($\hat{I}_{{{\text{TOT}}}} /A_{{{\text{INJ}}}}$ = 6.9%) classifications, using the QIBA threshold for the topical detector classifier. We note that this subject was converted to a true negative for the topical classifier when using the optimal threshold range.

## Discussion

The topical detector classifier had a significantly higher diagnostic performance for detecting extravasations compared to the radiologist. This demonstrates that leveraging topical detector semi-quantification of extravasation activity results in a robust classification scheme. The performance deficit in the radiological analysis may have been attributed partially to the dramatically elevated contrast of suspected extravasations, relative to normal physiological uptake. For instance, as demonstrated in Fig. 7A for a suspected extravasation classified as “severe” by the radiologist, although SUV max (~ 20) was comparable to the bladder, the normalized total activity in the injection area ($I_{{{\text{TOT}}}} /A_{{{\text{INJ}}}} =$ 0.7%) lead to a ground truth classification of “none.” This increased contrast can also make selecting optimal display windowing and leveling challenging. The topical detector classifier developed here, calibrated only on static PET images, has almost matched diagnostic performance (i.e., 80% sensitivity and 100% specificity) to a prior classifier [7] (i.e., 82% sensitivity and 100% specificity) developed from a qualitative assessment of dynamic PET TACs. Consequently, the use of the dynamic PET data and the full topical detector TACs may further improve the semi-quantitative topical detector classifier. Combining quantitative information and deep learning algorithms [18] for both the dynamic and static cases is expected to offer additional benefits to topical detector diagnostic performance. This retrospective analysis served as a hypothesis-generating study, and we hypothesize that the diagnostic performance advantage of the topical detector classifier relative to radiological review would be observed in a prospective study, as detailed in the discussion of limitations below.

The strong linear correlation between log-transformed ground truth and topical detector extravasation activity estimates (Fig. 4) demonstrated that the topical detector system provides semi-quantification of extravasation severity. This finding strengthens the key assumption: background signal from the body and arms is comparable in both topical detectors, such that the difference in TACs is primarily due to excess activity from an extravasation in the injection arm. As demonstrated by the linear regression analysis and multiclass confusion matrix (Table 1), however, both quantification and classification performance were reduced in some subjects. The limited detector number (i.e., two), use of uncollimated detectors, and absence of physics corrections (i.e., solid angle, scattered photons, and photon attenuation) can all decrease extravasation activity semi-quantification. These physics effects (primarily solid angle and lack of collimation) are further compounded and influenced by (1) the variable and unknown distance between topical detectors and extravasations, and (2) heterogeneity of extravasation tracer distributions. Such problems in nuclear medicine are well understood and in part have motivated the move away from planar scintigraphy to SPECT and PET. Overall, these limitations prevent 3d reconstruction and absolute quantification of extravasation activity uptake for the topical detector system. For instance, accounting for solid angle alone, the discrepancies in estimated extravasation activity may have been observed if the injection arm topical detector was placed closer to the relatively lower uptake extravasation in Fig. 6A compared to the effective subject averaged distance embedded in the linear regression models. Employing injection site-specific calibrations, of topical detector to ground truth PET data, may be one approach to compensate for this deficit. These semi-quantification limitations were also likely the sources of the heteroscedasticity observed in the linear regression of unaltered PET image extravasations versus topical detector rates (Fig. 4C). As the variance in topical detector rates as a function of extravasation activity was unknown, we utilized the log fit in (4) to suppress outliers and correct for heteroscedasticity, instead of weighted regression.

In addition, there were some cases where topical detector signal asymmetry was impacted by activity outside of the extravasation. As shown in Fig. 7B, suspicious uptake on the abdomen that was near the tracer extravasation may have contributed to the overestimation of topical detector extravasation activity. Any asymmetric normo- or pathophysiological uptake in arm tissue, or any tissue near a single detector, may also reduce the diagnostic performance of the topical detectors, for instance, due to arthritis [19]. However, these levels of FDG uptake are typically much lower than observed in the extravasations here, suggesting that such asymmetric uptake would largely impact the “none” to “minor” extravasation classes. We note that, although these limitations listed in the last two paragraphs likely had a substantial impact on topical detector semi-quantification and multiclass performance, addressing them is expected to result in a minor improvement in the binary classifier (primarily diagnostic sensitivity).

There were several limitations in our study. The comparatively low number of high activity extravasations (positive cases on the binary analysis) may have reduced statistical power in the diagnostic performance comparisons. Two factors may have contributed to this deficit: (1) the limited fraction of subjects with the injection site in the PET FoV, and (2) the relatively low number of severe extravasations, for this retrospective review from a single institution. To increase subject numbers, a multicenter prospective study could be applied that identifies subjects with extravasations, using the topical detectors, and then images the injection site of these subjects. The gap between topical detector measurements and the beginning of PET acquisitions (median difference of 15 min) was an additional limitation. This time difference required us to extrapolate topical detector TACs to estimate the count rate difference ($\hat{D}$) during PET. As shown in Fig. 3, the extrapolation algorithm showed comparatively high accuracy and precision, but baseline shifts occurring after the topical detector TAC cutoff can lead to bias. As the topical detectors are relatively small (i.e., 3 × 3 × 3 mm BGO crystals) and uncollimated, subtle changes in the uptake distribution near the injection detector (e.g., a displaced high uptake blood vessel caused by patient motion) could have produced these TAC artifacts. Ideally, topical detectors would remain in place and generate TACs up to the start of PET imaging. However, standard subject preparation (e.g., bladder voiding) and patient positioning make continuous topical detector measurements challenging and were not an option in this retrospective analysis. More robust extrapolation methods (e.g., deep learning) or a prospective human research study utilizing continuous topical detector TACs are possible alternatives. Radiotracer extravasation represents only one factor that impacts PET SUV measurements. Other biological (e.g., patient body size, blood glucose, uptake time) and technical factors (e.g., scan time, reconstruction parameters, etc.) can produce a similar to greater impact on SUV performance [20]. Thus, it is critical to minimize all of these potential sources of error to maximize the clinical performance of quantitative PET imaging.

The comparatively high diagnostic performance of the topical detector binary classifier for detecting extravasations, compared to the radiologist, may benefit both patient care and clinical operations. For instance, in therapy monitoring studies, if the technologist or radiologist suspects an extravasation then QIBA suggests imaging the extravasation site [6] to evaluate the study inclusion of the scan data. One option is to image and evaluate the injection site for all subjects (limitations discussed in the Background). However, the low specificity of the radiological analysis (Table 3) indicates that this approach would be unnecessary in more than 68% of cases, compared to relying on topical detector classification alone. Specifically, subjects with positive topical detector classification would undergo injection site imaging and more robust extravasation activity estimation with our PET ground truth algorithm, improving study data quality. Extravasations can produce significant radiation dose to the underlying tissue and skin exceeding regulatory limits [21]. The topical detector classifier would better limit dosimetric characterization to those subjects in need versus radiological analysis. For clinical care, if an extravasation exceeding 5% $A_{{{\text{INJ}}}}$ is detected, potentially negatively impacting SUV bias, then one outcome is to reschedule the subject for a repeat scan. Again, the limited specificity of the radiological analysis (Table 3) would result in an excessive number of unnecessary repeat studies compared to relying on the topical detector classifier alone.

## Conclusions

The topical detector system, calibrated using quantitative static PET measurements, provides semi-quantification of total extravasation activity. The resulting binary classifier offers significantly improved extravasation detection compared to qualitative radiological analysis. The ability to accurately detect extravasations before PET imaging could be used to improve human subject studies, patient care, and clinical operations.

## Supplementary Information

**Additional file 1.** Monte Carlo simulation of the topical detector response for an ideal tracer injection.

## Data Availability

The data that support the findings of this study are available on request from the corresponding author, SLB. The data are not publicly available due to them containing information that could compromise research participant privacy.

## Abbreviations

FDG: ^18^F-fluorodeoxyglucose
SUV: Standardized uptake value
QIBA: Quantitative imaging biomarker alliance
FoV: Field of view
TAC: Time-activity curve
VOI: Volume of interest
OSEM: Ordered-subsets expectation maximization
BGO: Bismuth germinate
PPV: Positive predictive value
NPV: Negative predictive value
MCC: Matthews correlation coefficient
