# Supplementary material for: Development of a classifier for [18F]fluorodeoxyglucose extravasation severity using semi-quantitative readings from topically applied detectors
Source: EJNMMI Phys. 2022 Sep 14;9:61. doi: 10.1186/s40658-022-00488-6 (PMC9474785; doi:10.1186/s40658-022-00488-6)
Supplement: Supplementary file 1 — Additional file 1. Monte Carlo simulation of the topical detector response for an ideal tracer injection. [file 40658_2022_488_MOESM1_ESM.docx]

Additional file 1 for: “Development of a Classifier for [18F]Fluorodeoxyglucose Extravasation Severity Using Semi-Quantitative Readings from Topically Applied Detectors”

**Monte Carlo simulation of the topical detector response for an ideal tracer injection**

Monte Carlo simulations were performed in order to investigate the expected output of Lara radiopharmaceutical injection monitoring detectors placed near the antecubital fossa for a hypothetical ideal injection (i.e., no extravasation). We used the ICRP adult male mesh-type reference phantom [1], which includes all source and target regions of interest for generalized transport and dosimetric investigations. The male phantom data represent an adult with height of approximately 1.75 m and mass of approximately 71 kg.

Assuming an 18F-FDG administration of 370 MBq, biodistribution ratios and radioactivity were assigned to each region of the phantom using values reported by Ramos et. al. [2]. Within each region, radioactivity was assumed to be uniformly distributed.

We created mesh files that realistically represent the detector geometry and arranged them in the phantom’s frame of reference relative to the arms as they would be during clinical use.

The GATE Monte Carlo simulation framework [3] version 9.1 was then used to calculate emission transport and absorption of back-to-back mono-energetic (511 keV) gamma ray emissions. Each region within the simulated geometry was assigned an appropriate material type to enable realistic consideration of attenuation and scatter. For example, all regions within the brain were assigned the GATE material “brain” and the scintillation crystals within each detector were assigned “BGO.” The previously determined radioactivity levels for each phantom source region were assigned within GATE, and the simulated timespan was set to 1 sec.

All events which resulted in energy being deposited in the crystal region of either detector were recorded and exported for analysis. Expected detector output was calculated from the simulation results using the published characteristics of the detector hardware [4].

Agreement between the two detectors was good with differences of less than 5% (409 cps for the right-arm and 390 cps for the left-arm). Figure S1 depicts the simulation geometry, including the detectors placed proximal to each antecubital fossa. Table S1 details the output for each detector by source organ, both in count rates and percent of total recorded by each detector.

**Table S1.** Detector output by source organ.

| **Source Organ** | **Right Arm** | | **Left Arm** | |
| --- | --- | --- | --- | --- |
|  | cps | % | cps | % |
| Adrenals | 0 | 0.0% | 0 | 0.0% |
| Bladder | 0 | 0.0% | 1 | 0.2% |
| Brain | 2 | 0.5% | 1 | 0.3% |
| Gall Bladder | 0 | 0.1% | 0 | 0.0% |
| Heart | 4 | 1.0% | 17 | 4.5% |
| Intestines | 14 | 3.3% | 31 | 7.8% |
| Kidney | 9 | 2.3% | 6 | 1.4% |
| Liver | 47 | 11.6% | 3 | 0.9% |
| Skeleton | 4 | 1.1% | 3 | 0.7% |
| Lungs | 4 | 0.9% | 1 | 0.4% |
| Pancreas | 1 | 0.1% | 0 | 0.1% |
| Spleen | 0 | 0.0% | 9 | 2.2% |
| Stomach | 0 | 0.1% | 7 | 1.9% |
| Thyroid | 0 | 0.0% | 0 | 0.0% |
| Rest of Body | 322 | 78.8% | 311 | 79.8% |
| **Total** | **409** | **100.0%** | **390** | **100.0%** |


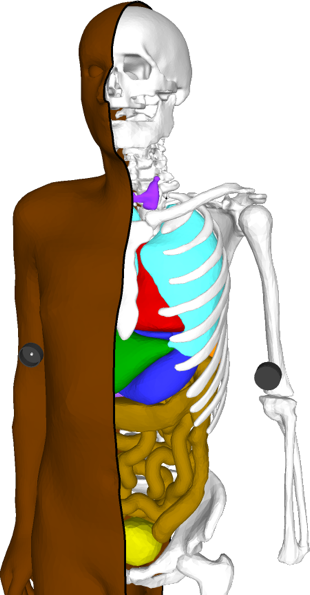


**Figure S1.** Digital anthropomorphic mesh phantom used for the simulation, including detectors in place proximal to each antecubital fossa.

**References**

1. Kim CH, Yeom YS, Petoussi-Henss N, Zankl M, Bolch WE, Lee C, et al. ICRP Publication 145: Adult Mesh-Type Reference Computational Phantoms. Ann ICRP. 2020;49:13-201. doi:10.1177/0146645319893605.

2. Ramos CD, Erdi YE, Gonen M, Riedel E, Yeung HW, Macapinlac HA, et al. FDG-PET standardized uptake values in normal anatomical structures using iterative reconstruction segmented attenuation correction and filtered back-projection. Eur J Nucl Med. 2001;28:155-64. doi:10.1007/s002590000421.

3. Jan S, Santin G, Strul D, Staelens S, Assie K, Autret D, et al. GATE: a simulation toolkit for PET and SPECT. Phys Med Biol. 2004;49:4543-61. doi:10.1088/0031-9155/49/19/007.

4. Knowland J, Lipman S, Lattanze R, Kingg J, Ryan K, Perrin S. Technical Note: Characterization of technology to detect residual injection site radioactivity. Med Phys. 2019;46:2690-5. doi:10.1002/mp.13536.
